# Application of integrated management bundle incorporating with multidisciplinary measures improved in-hospital outcomes and early survival in geriatric hip fracture patients with perioperative heart failure: a retrospective cohort study

**DOI:** 10.1007/s40520-021-02038-z

**Published:** 2022-01-24

**Authors:** Mingming Fu, Yaqian Zhang, Junfei Guo, Yuqi Zhao, Zhiyong Hou, Zhiqian Wang, Yingze Zhang

**Affiliations:** 1 Department of Geriatric Orthopedics, The Third Hospital of Hebei Medical University, Shijiazhuang, Hebei 050051 People’s Republic of China; 2 Department of Orthopaedic Surgery, The Third Hospital of Hebei Medical University, Shijiazhuang, Hebei 050051 People’s Republic of China; 3 NHC Key Laboratory of Intelligent Orthopeadic Equipment (The Third Hospital of Hebei Medical University), Hebei, People’s Republic of China; 4 Chinese Academy of Engineering, Beijing, 100088 People’s Republic of China

**Keywords:** Hip fracture, Geriatric, Perioperative heart failure, Complications, Survival, Management bundle

## Abstract

**Background:**

In elderly, hip fracture is often complicated by perioperative heart failure, related to worse prognosis. We aimed to analyze the effects of integrated management bundle incorporating with multidisciplinary measures on in-hospital outcomes and early survival in elderly hip fracture patients with perioperative heart failure.

**Methods:**

In this retrospective cohort study, a total of 421 hip fracture patients aged 65 and over who developed perioperative heart failure were included. According to different perioperative management modes applied, patients were retrospectively divided into multidisciplinary management group (Group A), including 277 patients, and integrated management bundle group (Group B), including 144 patients. The B-type natriuretic peptide (BNP) and C-reactive protein (CRP) levels, complications, length of stay, and hospitalization costs were observed and compared between two groups. Overall survival was compared by Kaplan–Meier methods. Cox regression analysis was used to identify prognostic factors associated with overall survival.

**Results:**

A total of 421 patients were enrolled for analysis, including 277 in Group A and 144 in Group B. BNP and CRP levels were significantly decreased compared with admission (*P* < 0.05). Furthermore, BNP and CRP in Group B decreased much more than those in Group A (*P* < 0.05). The reductions were observed in length of stay, hospitalization costs and incidence of pulmonary infection, hypoproteinemia, and acute cerebral infarction in Group B (all *P* < 0.05). The Kaplan–Meier plots showed significantly superior overall survival in Group B. Integrated management bundle was independent favorable prognostic factors.

**Conclusions:**

The integrated management bundle incorporating with multidisciplinary measures significantly improved the therapeutic effect of perioperative heart failure, reduced inflammatory response, and yielded better hospital outcomes. It brought better survival benefits for geriatric hip fracture patients with perioperative heart failure. The results of this study can play an important role in clinical work and provide a valuable theoretical basis for selection of management model in elderly hip fracture patients with perioperative heart failure.

## Introduction

Due to rapidly aging populations, the number of elderly hip fracture patients is increasing [1, 2]. Hip fractures commonly result in loss of functionality, disability, and increase mortality in elderly, which has become a global medical and health problem [3]. In such case, effective interventions are needed to improve the function and prognosis of elderly hip fracture patients. A recent study by Invernizzi et al. [4] showed that a multidisciplinary rehabilitative could improve function and reduce disability in hip fracture patients. Gamboa-Arango et al. [5] reported that better functionality at the hospital discharge improved the prognosis of elderly hip fracture patients. Trevisan et al. [6] believed enhanced patient management, aggressive rehabilitation might further reduce mortality of elderly hip fracture patients.

Heart failure is one of the most frequent perioperative complications in elderly patients with hip fractures and an important risk factor for mortality after hip surgery [7–12]. The mechanism of perioperative heart failure is extremely complex. Geriatric hip fracture patients are in a state of persistent stress, because of fear, pain, anxiety, and blood loss, leading to increased myocardial oxygen consumption and imbalance between myocardial oxygen supply and demand, followed by perioperative myocardial ischemia and injury. This condition, when severe, usually leads to myocardial stunning and heart failure [13–15]. Due to the prevalence and complexity of heart failure, perioperative heart failure management has become a major challenge for physicians [16, 17]. Although much attention has been paid to the perioperative management in elderly hip fracture patients, further research is needed to explore a better management model in elderly hip fracture patients with perioperative heart failure. On the basis of clinical practice and experiences, we find that integrated management bundle incorporating with multidisciplinary measures is more suitable for elderly hip fracture patients with perioperative heart failure.

We aimed to analyze the effects of integrated management bundle incorporating with multidisciplinary measures on in-hospital outcomes and early survival in elderly hip fracture patients with perioperative heart failure. We hypothesized that this new type of management model was associated with lower B-type natriuretic peptide (BNP) level, lower complication rates, fewer total hospitalization costs, shorter hospital length of stay, and better survival.

## Material and methods

### Patients and groups

The retrospective cohort study was based on data collected from Jan, 2017, to Sept, 2019, at a single Level I trauma center in China. The study protocol was approved by the institutional review board of the third Hospital of Hebei Medical University, and an exemption from the informed consent was obtained. Inclusion criteria were hip fracture patients aged 65 years and over with perioperative heart failure, who had a delay less than 1 week from injury to admission, and underwent hip surgery. Diagnosis of heart failure should be based on clinical signs, symptoms, and prior cardiovascular history and further confirmed by appropriate additional investigations, such as BNP, electrocardiogram, chest X-ray, and echocardiography [18]. Besides, all patients enrolled were treated with common drugs for heart failure, and blood samples were collected at least right after admission as well as just before discharge to determining BNP and C-reactive protein (CRP). Exclusion criteria were multiple fractures or injuries, pathological fractures, chronic heart failure, malignancy, autoimmune disease, and preexisting systemic inflammatory comorbidity. The patients who admitted to our department from Jan 2017 to Sept 2019, and met the inclusion criteria, with no exclusion criteria, were included in this analysis. According to different perioperative management modes applied, patients were retrospectively divided into multidisciplinary management group (Group A) and integrated management bundle group (Group B).

### Perioperative management

Different from most hospitals, our hospital has a specialist ward for geriatric orthopedics staffed by orthopedists, internists, rehabilitation physicians, and trained nursing personnel, that provides centralized management and sustainable 24/7/365 geriatric support. The multidisciplinary team for geriatric fracture participates in the ward round 7 days a week. The patients are assessed by at least two orthopedists who lead the team and make the preparation related to the surgical procedure, along with an internist who is available to review perioperative care for patients with comorbidities, in cooperation with an anesthetic consultant. Rehabilitation physicians guide the patients in rehabilitation care. In the first stage, multidisciplinary management was applied to elderly hip fracture patients with perioperative heart failure.

Depending on clinical experience and related guidelines, the team optimized the original protocol, proceeding to a new stage of perioperative management, which is the second stage. In the second stage, considering the characteristics of the elderly hip fracture patients and the unique medical system in China, we simplified, optimized, and integrated the existing management model to form the integrated management bundle incorporating with multidisciplinary measures, so that it is more in line with the clinical actual situation and more feasible. The core members of the team include orthopedic specialists, internal medicine specialist with well-recognized skills in geriatric medicine, anesthesiologists, rehabilitation physicians, and specialist nurses. From the time of hospital admission onwards, hip fracture patients were given the integrated management bundle, including monitoring, evaluation and education, respiratory management, volume management, nutritional support, blood and thrombosis management, sedation and analgesia, and tube management. The internist with well-recognized skills in geriatric medicine focused on the comprehensive assessment of diseases in multiple systems on the basis of a holistic view, without relying on clinical consultations. The specific measures were described in more detail below.

Group A was in the first stage. In the course of multidisciplinary management, the monitoring of electrocardiogram, mean arterial pressure, and pulse oxygen saturation was performed in patients with severe comorbidities, which guaranteed the detection and management of cardiovascular and cerebrovascular complications promptly [19, 20]. Oxygen treatment was restricted to patients with pulmonary infection or respiratory failure. Assessment of nutritional status was not routinely recommended, only if the patient recently lost weight or had a low body mass index on admission. Nutrition therapy was only available for a subset of patients. Food was not allowed to take within 8 h before surgery. The analgesics, including either an opioid, a nonsteroidal anti-inflammatory drug (NSAID), or acetaminophen, were applied to ease pain. Low-molecular-weight heparin and ankle pump exercise were administered to prevent deep vein thrombosis. Patients suspected of urinary retention were given single catheterization. Indwelling time of urethral catheter took for several days, in case of persistent urinary retention [21].

Group B was in the second stage. Patients in Group B were applied to integrated management bundle incorporating with multidisciplinary measures, including monitoring, evaluation and education, respiratory management, volume management, nutritional support, blood and thrombosis management, sedation and analgesia, and tube management. The monitoring of electrocardiogram, mean arterial pressure, and pulse oxygen saturation was suitable for all patients. Once admitted, patients were assessed by comprehensive geriatric assessment [22], which could detect potential risks and prompt intervention immediately. The health care should be effectively preached and there was an opportunity to establish harmonious relationship with patients and their families. Respiratory management was listed as follows: chest physiotherapy and breathing exercises were required, including actively cough, accessary posture productive cough, and turnover [23]. Low-flow inhale oxygen and atomization were indispensable measures. The patient was treated with nebulization of salbutamol sulfate, ipratropium bromide, and budesonide twice daily. Patients had a documented order for strict monitoring of fluid intake and output, to achieve the negative fluid balance [24]. All of the patients were evaluated for nutritional status on admission, and nutrition therapy was started in accordance with specific conditions [25]. Probiotics and prokinetic agents were used to avoid acute gastrointestinal dysfunction. Oral feeding was the preferred method, and if nutrient intake was insufficient, early detaining gastric tube was chosen to avoid electrolyte imbalance. We recommended that patients maintained a hemoglobin level of at least 10 g per deciliter of blood. The basic, physical, and pharmacological prevention measures were actively applied to prevent the deep vein thrombosis of lower extremities [26]. For perioperative agitation and delirium, attempts should be made to identify and relieve the likely causative triggers, beyond just those drug treatments. Pain management focused on the use of multimodal analgesia, which included effective early analgesia, the application of acetaminophen and nonsteroidal anti-inflammatory medication, and patient-controlled analgesia [27]. Patients suspected of urinary retention were given ultrasound bladder scan. Urinary retention was treated with a single catheterization at first, and the second remained urethral catheter in place for 1–2 days[21].

### Data collection

Clinical data and baseline variables of all patients were collected from the patients’ electronic medical records. The following information was extracted: gender, age, mechanisms of injury, comorbidities, fracture type (femoral neck fracture or intertrochanteric fracture), surgery type (fixation or replacement), anesthesia type (general or regional), intraoperative blood loss, duration of operation, complications, BNP (in units of pg/mL), CRP (in units of mg/L), admission delay time, hospital length of stay, and total hospitalization costs. The follow-up started at the date of enrollment in the cohort and the endpoint was the date of death or the end of the study, whichever came first. The primary outcomes included BNP measured before discharge and overall survival at 2 years. The secondary outcomes included the rates of other perioperative complications, hospital length of stay, and total hospitalization costs.

C-reactive protein is one of the most representative markers in the acute phase of systemic inflammatory response, which has been found to be associated with coronary heart disease and heart failure [28, 29]. Whether there was a correlation between CRP and BNP in elderly hip fracture patients with perioperative heart failure has not been studied. Moreover, it was convenient to dynamically monitor CRP levels clinically. Thus, we used CRP as inflammatory marker.

### Statistical analysis

The continuous variables were evaluated for normality by using the Shapiro-Wilk test. Normally distributed variables were presented as mean and standard deviation (SD); otherwise, they are presented as median and interquartile range. Categorical variables were presented as numbers and percentages. Student’s *t*-test or Mann–Whitney *U* test was used to compare differences between groups for continuous variables as appropriate, while the Chi-square test or Fisher exact test for categorical variables. The correlation of BNP and CRP was evaluated by Spearman’s correlation and statistical significance. Kaplan–Meier method was used to estimate the survival, and any difference in survival was evaluated with a log-rank test. Univariate and multivariate analyses of survival outcomes were conducted with the Cox proportional hazards regression model, to determine the independent prognostic factors. All the statistical analyses and graphics were performed with the SPSS statistical software (version 26.0) and R statistical software (version 3.6.3). The level of significance was set at *P* < 0.05.

## Results

### Patient characteristics

**Fig. 1:**
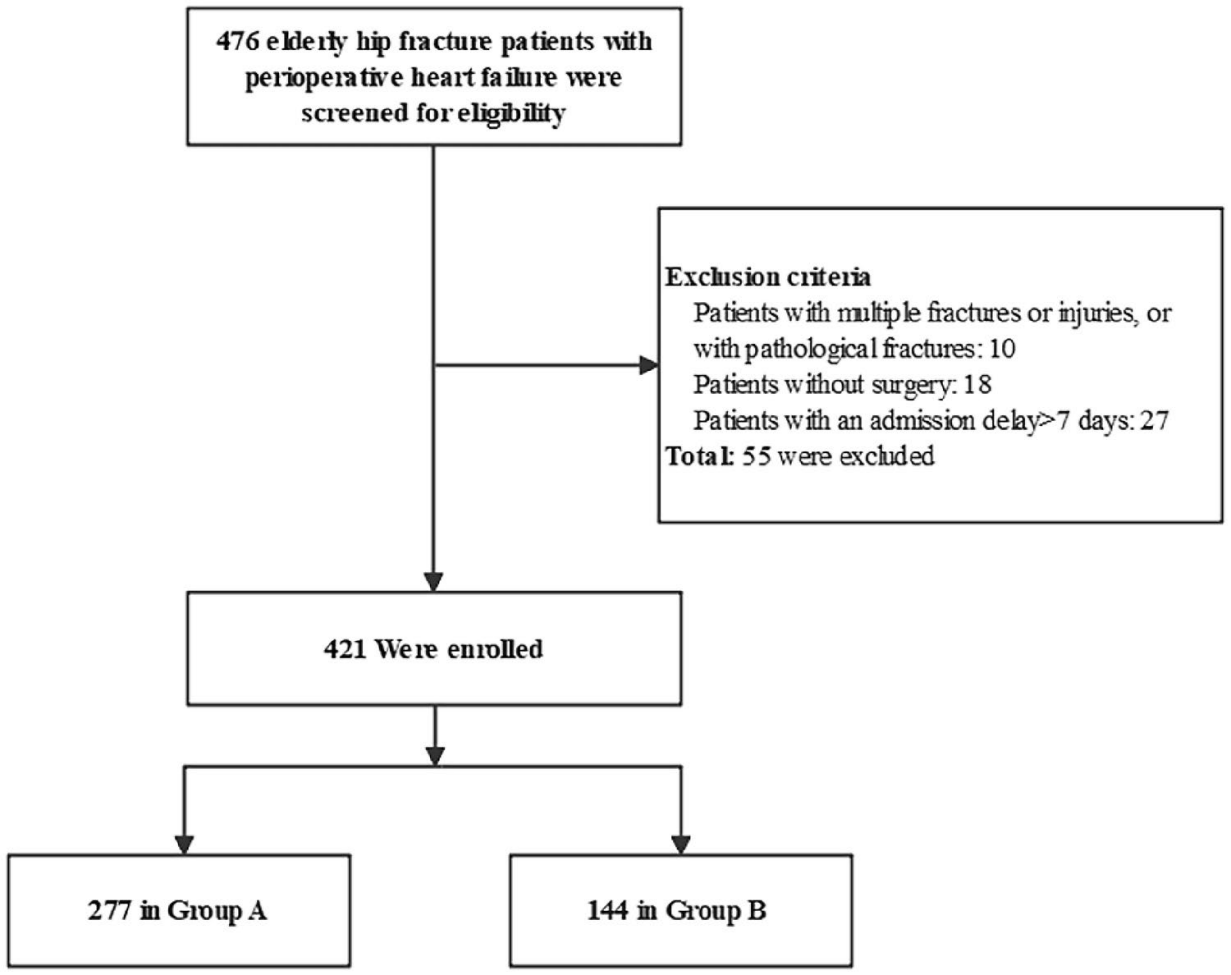
The flow diagram of this study

From Jan 2017 to Sept 2019, a total of 476 geriatric patients with hip fracture were screened to participate in this study. Among them, 55 patients were eliminated by the exclusion criteria, and the remaining 421 were finally analyzed. Specifically, 10 patients had multiple fractures or injuries, or pathological fractures; 18 patients received nonsurgical treatment; 27 patients admitted with a delay of more than 7 days (Fig. 1). A total of 421 patients met inclusion and were included in our analysis, of which 277 received multidisciplinary management and 144 underwent integrated management bundle. A majority of patients was female (70.8%) and mean age was 81.8 years (SD 7.1). The average follow-up was 21 months. The clinical characteristics of patients in two groups are presented in Table 1, which did not differ significantly.

**Table 1:** Baseline characteristics of geriatric hip fracture patients with perioperative heart failure

	Total (*n* = 421)	Group A (*n* = 277)	Group B (*n* = 144)	*χ*^2^/*t*/*Z*	*P* value
Gender, *n* (%)					
Male	123 (29.2%)	80 (28.9%)	43 (29.9%)	0.044	0.834
Female	298 (70.8%)	197(71.1%)	101 (70.1%)		
Age, mean ± SD (years)	81.8 ± 7.1	81.8 ± 7.4	81.7 ± 6.5	0.192	0.848
Age group, *n* (%)					
< 80 years	151 (35.9%)	100 (36.1%)	51 (35.4%)	0.019	0.890
≥ 80 years	270 (64.1%)	177 (63.9%)	93 (64.6%)		
BMI (normal/overweight/obesity)	272/115/34	177/76/24	95/39/10	0.414	0.813
Mechanism of injury, *n* (%)					
Low energy	408 (96.9%)	265 (95.7%)	143 (99.3%)	3.062	0.080
High energy	13 (3.1%)	12 (4.3%)	1 (0.7%)		
Fracture types, *n* (%)					
Femoral neck fractures	193 (45.8%)	131 (47.3%)	62 (43.1%)	0.685	0.408
Intertrochanteric fractures	228 (54.2%)	146 (52.7%)	82 (56.9%)		
Comorbidities, *n* (%)					
Hypertension	214 (50.8%)	146 (52.7%)	68 (47.2%)	1.141	0.286
Coronary heart disease	121 (28.7%)	83 (30.0%)	38 (26.4%)	0.591	0.442
Diabetes	92 (21.9%)	62 (22.4%)	30 (20.8%)	0.133	0.715
Cerebrovascular disease	157 (37.3%)	106 (38.3%)	51 (35.4%)	0.329	0.566
Surgical type, *n* (%)					
Replacement	183 (43.5%)	125 (45.1%)	58 (40.3%)	0.906	0.341
Fixation	238(56.5%)	152 (54.9%)	86 (59.7%)		
Anesthesia type, *n* (%)					
General	201 (47.7%)	133 (48.0%)	68 (47.2%)	0.024	0.877
Regional	220 (52.3%)	144 (52.0%)	76 (52.8%)		
Intraoperative blood loss	200 (200, 300)	200 (200, 300)	200 (200, 300)	− 0.568	0.570
Duration of operation	108.6 ± 30.8	110.0 ± 30.2	105.9 ± 31.8	1.294	0.196
Admission BNP	304 (233, 444)	290 (225, 457)	327 (249, 427)	1.515	0.130
Admission CRP	56.1 (37.4, 84.2)	56.3 (36.8, 81.6)	55.7 (38.5, 88.3)	0.360	0.719

Values are presented as mean ± standard deviation, median (P25, P75), or number (percentage) as appropriate. *SD* standard deviation, *BMI* body mass index, *BNP* B-type natriuretic peptide, *CRP* C-reactive protein. *χ*^2^ value is Chi-square test statistic, and *t* value is *t*-test statistic. *Z* is the *Z*-score for Wilcoxon test

### Comparison of complications and hospital outcomes

The comparison of perioperative complications and hospital outcomes is shown in Table 2. In terms of type of complications, Group B had a reduction in incidence of pulmonary infection, acute cerebral infarction, and hypoproteinemia (*P* = 0.023, *P* = 0.022, and *P* = 0.009, respectively). No differences were found in the incidence of arrhythmia, electrolyte disturbance, deep venous thrombosis of the lower limbs, and urinary infection between two groups. BNP and CRP values for both the two groups decreased postmanagement (*P* < 0.001). There was a greater decrease of BNP and CRP values in Group B as compared to Group A (*P* = 0.011, and *P* = 0.010, respectively). Moreover, integrated management bundle could shorten hospital length of stay and reduce total hospitalization costs than routine management (Table 2).

**Table 2:** Comparisons of perioperative complications and hospital outcomes between two groups

Variables	Group A (*n* = 277)	Group B (*n* = 144)	χ^2^/*t*/*Z*	*P* value
Perioperative complications				
Pulmonary infection	120 (43.3%)	46 (31.9%)	5.135	0.023*
Arrhythmia	96 (34.7%)	49 (34.0%)	0.017	0.897
Acute cerebral infarction	14 (5.1%)	1 (0.7%)	5.241	0.022*
Hypoproteinemia	212 (76.5%)	93 (64.6%)	6.779	0.009*
Electrolyte disturbance	209 (75.5%)	104 (72.2%)	0.518	0.472
Deep venous thrombosis of the lower limbs	134 (48.4%)	63 (43.8%)	0.814	0.367
Urinary infection	6 (2.2%)	7 (4.9%)	1.487	0.233
Discharge BNP	162 (98, 273)	128 (94, 191)	− 2.547	0.011*
Discharge CRP	31.5 (16.0, 49.6)	25.8 (14.1, 39.1)	− 2.589	0.010*
Length of stay	15.1 ± 5.3	13.2 ± 4.9	3.416	0.001*
Total hospitalization costs	7.2 ± 2.0	6.7 ± 2.0	2.606	0.010*

Values are presented as mean ± standard deviation, median (P25, P75), or number (percentage) as appropriate. *Statistically significant. *BNP* B-type natriuretic peptide, *CRP* C-reactive protein. *χ*^2^ value is Chi-square test statistic, and *t* value is *t*-test statistic. *Z* is the *Z*-score for Wilcoxon test

### The correlations between BNP and CRP levels

**Fig. 2:**
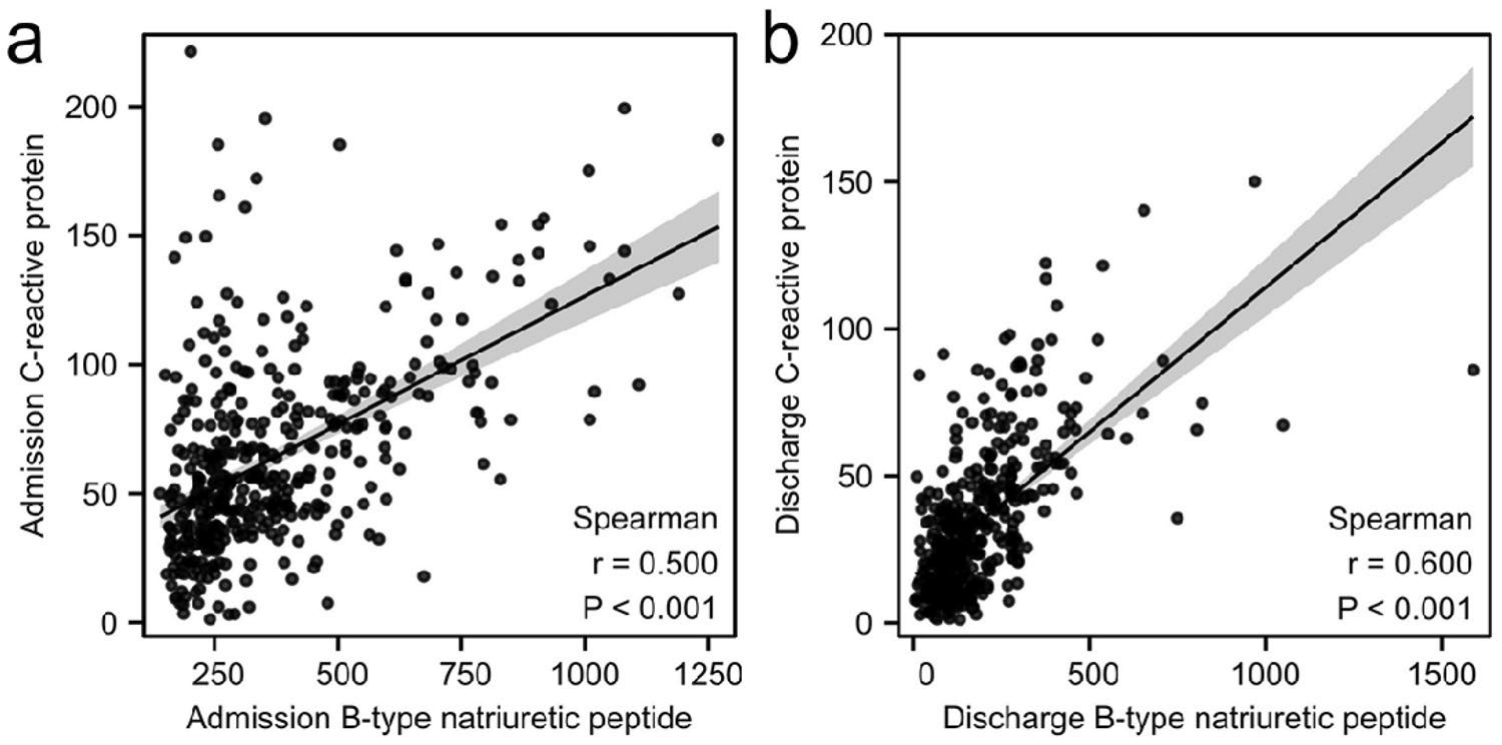
Scatterplot of the correlations between B-type natriuretic peptide and C-reactive protein levels

Scatterplot presenting the correlations between BNP and CRP levels is shown in Fig. 2. There was a moderate positive correlation between admission BNP and admission CRP levels (*r* = 0.500, *P* < 0.001). Likewise, Spearman correlation between discharge BNP and discharge CRP showed a moderate positive correlation (*r* = 0.600, *P* < 0.001).

### Survival analysis for patients

**Fig. 3:**
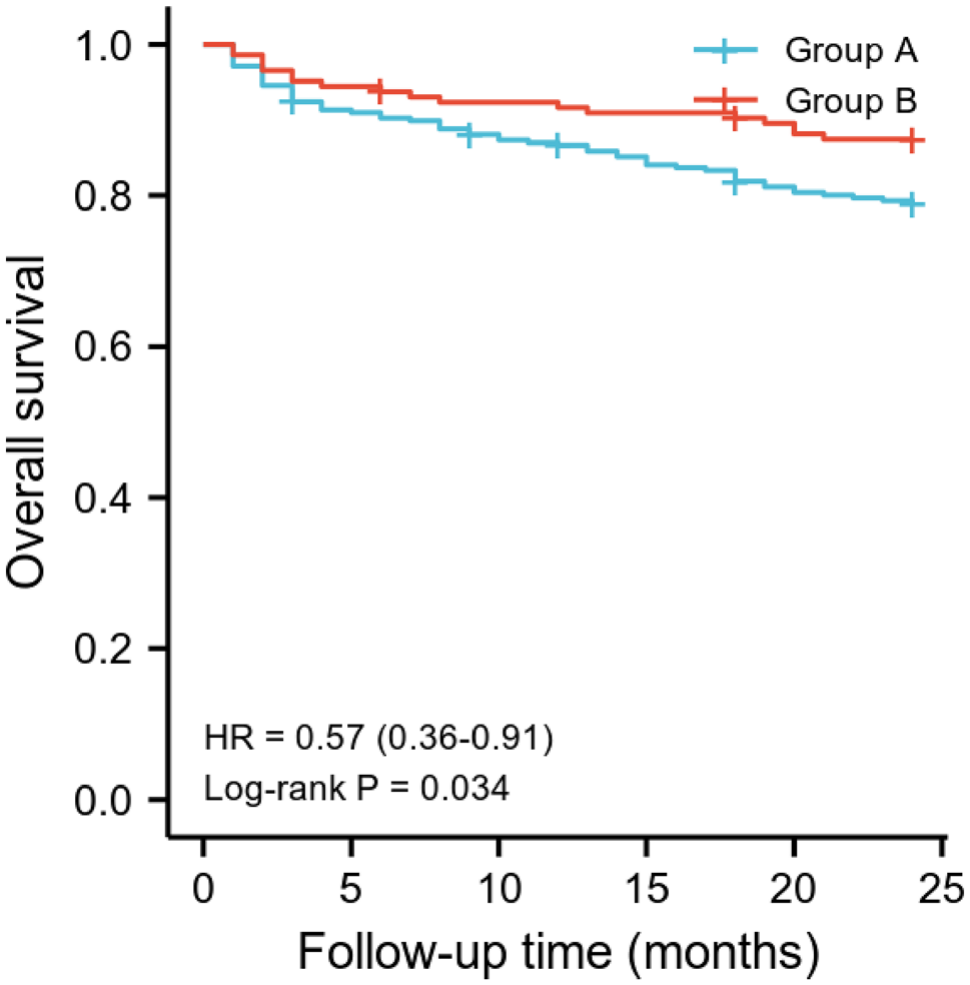
Kaplan–Meier survival curves for elderly patients with perioperative heart failure

Compared with multidisciplinary management group, the 2-year mortality was significantly lower in integrated management bundle group (20.9% vs 12.5%, *P* = 0.033). Kaplan–Meier survival curves for two groups are shown in Fig. 3. A comparison of survival curves using log-rank test indicated that integrated management bundle significantly improved survival (*P* = 0.034).

### Identify prognostic factors

We employed Cox proportional hazards model to analyze possible prognostic factors associated with overall survival. In univariate Cox model, the significant variables were age ≥ 80, comorbidities ≥ 2, and received integrated management bundle (*P* < 0.05). Multivariate analysis revealed age ≥ 80 and comorbidities ≥ 2 were independent risk factors for poor prognosis (HR > 1, *P* < 0.05). On the contrary, integrated management bundle was independent favorable prognostic factors (HR < 1, *P* < 0.05) (Table 3).

**Table 3:** Cox proportional hazards regression model for overall survival

Variables	Univariate Cox		Multivariate Cox	
	HR (95% CI)	*P* value	HR (95% CI)	*P* value
Gender				
Female	Reference	0.894	Reference	0.649
Male	1.034 (0.634–1.687)		1.121 (0.684–1.838)	
Age group				
< 80 years	Reference	0.007*	Reference	0.003*
≥ 80 years	2.090 (1.218–3.584)		2.331 (1.337–4.063)	
Mechanism of injury				
Low energy	Reference	0.779	Reference	0.924
High energy	0.818 (0.201–3.331)		0.933 (0.225–3.871)	
Comorbidities				
0–2	Reference	0.018*	Reference	0.008*
> 2	1.808 (1.108–2.949)		1.963 (1.196–3.224)	
Fracture type				
Femoral neck	Reference	0.692	Reference	0.065
Intertrochanteric	0.913 (0.582–1.432)		0.392 (0.145–1.058)	
Surgery type				
Replacement	Reference	0.865	Reference	0.125
Fixation	1.040 (0.660–1.640)		2.189 (0.804–6.010)	
Anesthesia type				
Regional	Reference	0.133	Reference	0.231
General	0.689 (0.435–1.092)		0.753 (0.473–1.199)	
Management mode				
Multidisciplinary management	Reference	0.037*	Reference	0.047*
Integrated management bundle	0.570 (0.336–0.968)		0.581 (0.341–0.992)	

*Statistically significant

## Discussion

In this retrospective study, we analyzed the clinical characteristics and prognosis of geriatric hip fracture patients with perioperative heart failure. In our results, the application of integrated management bundle incorporating with multidisciplinary measures had been found to be associated with lower BNP and CRP levels, lower complication rates, including pulmonary infection, acute cerebral infarction, and hypoproteinemia, fewer total hospitalization costs, shorter length of stay, and better survival. There was a moderate positive correlation between BNP and CRP levels.

Hip fracture patients are often of advanced age and suffer from multiple comorbidities [30], which poses challenges for clinical diagnosis and treatment. As a result, orthogeriatric management model comes into existence, which is the multidisciplinary collaboration model for older patients with orthopedic disorders. So far there are some consensus and established protocols about orthogeriatric management [27, 31–33]. In combination with the characteristics of the elderly hip fracture patients and the unique medical system in China, the management model is simplified, optimized, and integrated to form the integrated management bundle incorporating with multidisciplinary measures, so that it is more in line with the clinical actual situation and more feasible. Heart failure is one of the common and serious perioperative complications in elderly hip fracture patients [34]. Nevertheless, high-quality studies are still scarce, which is about the management on geriatric hip fracture patients with perioperative heart failure.

It is difficult to make an early diagnosis of heart failure based on symptoms and signs alone, which are often unspecific and similar to those of other diseases. In such clinical situations, BNP is often superior to clinical diagnosis for diagnosing heart failure [16]. Before discharge, BNP and CRP levels were significantly decreased compared with admission. Furthermore, BNP and CRP levels in integrated management bundle group decreased much more than that of multidisciplinary management group. The mechanism of perioperative heart failure is extremely complex. Specifically, stress triggers the activation of sympathetic nervous system, which mediates the release of catecholamines and glucocorticoids, increasing sodium retention, vasoconstriction, and cardiac workload, and ultimately leading to perioperative myocardial ischemia and injury. Another possible mechanism is that an abundant increase in inflammatory factors caused by stress leads to perioperative myocardial ischemia and injury. As diagnostic and prognostic markers of heart failure [35], brain natriuretic peptide (BNP) is secreted from the heart in response to stretch and stress. Raised levels of BNP positively correlate with the severity of heart failure [36–38]. C-reactive protein is a nonspecific indicator of the inflammatory response, applied to assess the severity of traumatic stress.

Medication remains the cornerstone of therapy for heart failure. Nevertheless, application of high-quality and effective management interventions plays a crucial role in promoting recovery and improving outcomes based on pharmacotherapy. According to the results of this study, the reduction was also observed in incidence of pulmonary infection, hypoproteinemia, and acute cerebral infarction in integrated management bundle group. We highlight the bundled application of numerous measures, especially respiratory management, volume management, nutritional support, and blood management in integrated management bundle group, which reduced traumatic stress and inflammatory responses much more than those in multidisciplinary group.

Recently, Zhang et al. discovered that the levels of plasma BNP were positively correlated with CRP levels in patients with sepsis [39]. A similar phenomenon was also observed in the present study. Various studies have shown that inflammatory response plays an important role in pathogenesis of heart failure, associated with the severity of traumatic stress. C-reactive protein is a nonspecific indicator of the inflammatory response, influenced by many factors, including surgical trauma, fracture, infection, and tumor [40–42]. In this study, we excluded patients with systemic inflammatory diseases and malignant tumors, to minimize other factors of influence on our results. Our study was a direct demonstration of correlation between perioperative heart failure and inflammation response in geriatric hip fracture patients. Nevertheless, the prognostic and diagnostic value of CRP required further investigation in geriatric hip fracture patients with perioperative heart failure.

The results showed that the integrated management bundle exerted significant survival benefits for geriatric hip fracture patients with perioperative heart failure in overall survival curves. Multivariate Cox regression analysis determined integrated management bundle was independent favorable prognostic factors. Neuerburg et al. [43] demonstrated that interdisciplinary orthogeriatric management improved the long-term outcome of hip fracture patients. Liu and Rosas et al. [25] presented clinical evidence demonstrating that enhanced recovery after surgery (ERAS) was associated with significant decreases in hospital length of stay and postoperative complication and it is effective in improving outcomes in surgical populations. That is to say, optimization of original perioperative management mode could improve outcomes of patients [20, 44–46]. Further studies should be carried out in order to better clarify the effect. These bundled measures should be considered as first steps in the development of a more polished set of measures, and studies on perioperative management have never ceased.

## Limitations and strengths

There are several limitations in this study. It is a single-center, retrospective cohort study and the intrinsic limitation of design seems to be inescapable. There is likely to be a selection bias due to lack of randomization. The variables involved in this study are limited to previously collected data. Moreover, the limited statistical power caused by small samples and short follow-up periods implies that the results should be interpreted with caution. The absence of standardization of the drugs administered among the elderly hip fracture patients with perioperative heart failure could be also viewed as a limitation of this study. Despite these limitations, this study has areas of strength. We provide clues on perioperative management of elderly hip fracture patients with perioperative heart failure.

## Conclusions

The integrated management bundle incorporating with multidisciplinary measures significantly improved the therapeutic effect of perioperative heart failure, reduced perioperative inflammatory response, and yielded better hospital outcomes. It brought better survival benefits for geriatric hip fracture patients with perioperative heart failure. The results of this study can play an important role in clinical work and provide a valuable theoretical basis for selection of management model in elderly hip fracture patients with perioperative heart failure.

## Data Availability

The data used to support the findings of this study are available from Zhiqian Wang upon request.
